# Energy balance during two days of continuous stationary cycling

**DOI:** 10.1186/1550-2783-4-15

**Published:** 2007-10-31

**Authors:** Ian B Stewart, Kelly L Stewart

**Affiliations:** 1 Institute of Health and Biomedical Innovation, Queensland University of Technology, Australia; 2 School of Public Health, Queensland University of Technology, Australia

## Abstract

This study examined the capabilities of an ultraendurance athlete to self-regulate their diet during an attempt on the record for the longest period of stationary cycling. The attempt required the athlete to complete at least 20 km/hr, with a 15 minute break allowed every eight hours. Laboratory tests determined a heart rate-oxygen consumption regression equation enabling calculation of energy expenditure from heart rate during the attempt. Energy intake was determined by a non-weighed dietary record collected at the time of consumption. The athlete completed 46.7 hours, covering 1126 km, at a speed of 24 ± 1.6 km/hr. He expended 14486 kcal and consumed 11098 kcal resulting in an energy deficit (-3290 kcal) and a weight loss (-0.55 kg). The carbohydrate (42 ± 32 g/hr), water (422 ± 441 ml/hr), and sodium (306 ± 465 mg/hr) intake were all below current recommendations. The athlete was unable to self-regulate his diet or exercise intensity to prevent a negative energy balance.

## Background

Ultraendurance athletes' are challenged in attempting to balance the high energy requirements of their sport with the high energy consumption required to compete successfully. When the energy intake exceeds 4500 kcal/d, the athlete will commonly have to ingest and digest food while actually competing [1]. In order to meet these energy requirements while competing and prevent abdominal distress and diarrhoea, the athlete typically selects energy dense foods, gels and liquids [1]. These types of foods allow gastric volumes to be kept to a comfortable level and dehydration to be prevented [2,3]. An exercise-induced suppression of appetite, in combination with a nibbling eating pattern of continuous snacking, often leads to an insufficient energy intake during prolonged exercise [4]. Disciplined eating by athletes plus appropriately prescribed diets by sports dietitians are required for the athlete to prevent a negative energy balance and its associated detrimental influence on prolonged endurance performance.

Few athletes have the discipline required to successfully self regulate their energy intake during ultraendurance exercise, but one previous casestudy occurred during the 1000 km running race from Sydney to Melbourne in Australia. Greek ultra marathoner, Yiannis Kouros', self regulated diet across the five days was almost entirely comprised of carbohydrate (95%). Energy dense Greek sweets and honey soaked biscuits were snacked on every 30 minutes, enabling Kouros to consume on average an amazing 11,074 kcal/d [5].

The purpose of this case study was to assess the capabilities of an experienced ultraendurance athlete to appropriately self regulate his energy intake during an attempt on the world record for the longest period of stationary cycling.

## Methods

### Subject

The subject was a 35 year old male amateur cyclist (height 172 cm; weight 72 kg) with previous experience in ultraendurance events. The subject gave informed consent to collect data during the record attempt.

### Subject preparation

The subject completed an intense program of endurance training spanning 6 months. He was provided with guidelines about the amounts and types of foods/fluids to consume throughout the world record attempt with specific emphasis on carbohydrate, sodium and fluid amounts. The subject was then responsible for planning and preparing the appropriate items for the ride. He began the attempt with access to ample sports drinks, protein bars and energy gels (Endura™).

### Pre-record attempt laboratory testing

The athlete completed two cycle ergometer protocols a week prior to the record attempt. The first comprised four submaximal workloads, commencing at 0 W and increasing by 25 W every 2.5 minutes. This test was conducted to develop a relationship between heart rate and oxygen uptake (VO_2_) in order to estimate energy expenditure during the record attempt [6]. The second cycle protocol was an incremental (30 W/min) test to exhaustion to determine aerobic capacity (VO_2_max), ventilatory threshold, and peak power.

All testing was undertaken on a cycle ergometer (Excalibur Sport, Lode, Gronigen, The Netherlands) with ventilation and expired gas collected and analysed every 15 seconds using a metabolic cart (Moxus, AEI Technologies, Pennsylvania, USA). Oxygen consumption (VO_2_), minute ventilation (V_E_), carbon dioxide production (VCO_2_), and respiratory exchange ratio (RER) were calculated. Before testing, the gas analysers were calibrated with a known gas mixture and room air. Heart rate was measured by telemetry (Polar Vantage XL, Kemple, Finland) and recorded every 15 seconds.

The VO_2 _and heart rate values obtained during the multiple submaximal workload test were averaged over the final minute of each of the four workloads and a linear regression was fitted to the data. Standard indicators for achieving VO_2 _max were applied to the incremental test to exhaustion: volitional fatigue; a plateau in oxygen consumption with increasing work rate; heart rate ≥ 90% of age predicted maximum; and a respiratory exchange ratio ≥ 1.15. Given that three of the preceding criteria were met, values for maximal oxygen consumption were determined by averaging the four highest consecutive 15 second values.

### World record attempt

The previous world record stood at 77 hours and 15 minutes. The attempt required the athlete to complete at least 20 km per hour, with a 15 minute break off the bike allowed every eight hours. The record was attempted on a stationary cycle ergometer (Tempo, Giant, Australia). The distance covered and the speed maintained was calculated by a calibrated bike computer (VELO5, Cat Eye, Japan). Throughout the record attempt, the athlete's heart rate (s610i, Polar Electro Oy, Kempele, Finland) and blood pressure was monitored, and the self regulated fluid and food intake recorded. Heart rate was recorded every minute and energy expenditure calculated from the equation obtained from the laboratory submaximal workload test. Blood pressure was recorded every hour. Body mass was measured every eight hours and urine output was weighed throughout. The total energy, carbohydrate, protein, fat and sodium consumed by the athlete was analysed using a dietary software program (Foodworks Professional Version 4.00, Xyris Software, Australia).

Throughout the attempt the athlete was provided with verbal encouragement and physical assistance to continue eating and drinking and encouragement to continue cycling.

## Results

### Laboratory testing

The subject's VO_2 _max from the incremental test was 60.3 ml/kg/min and he achieved a peak power of 315 W. The relationship between heart rate and VO_2 _determined with the multiple submaximal workloads was represented by the following equation (VO_2 _(ml/min) = 26.826 * heart rate – 1434.6, r^2 ^= 0.9974). The energy expenditure measured in kcal was calculated by multiplying the estimated VO_2 _(in L/min) by 4.948. This conversion factor was calculated from the average non-protein respiratory quotient measured during the multiple submaximal workloads [7].

### Record attempt

The athlete completed 46 hours 44 minutes and 20 seconds of continuous stationary cycling during the record attempt. The ambient temperature ranged from 16.9 to 27.9°C and the relative humidity from 44 to 65%. The total distance covered was 1126 km at an average speed of 24 ± 1.6 km/hr and heart rate of 91 ± 15 bpm (Table 1). The macronutrient intake, total energy intake and expenditure, averaged over eight hour periods of continuous cycling, are presented in Table 2. Average hourly ingestion rates were carbohydrate 42 g (range 0–125), fat 5 g (range 0 – 34), protein 6 g (range 0 – 28), and sodium 306 mg (range 0 – 2781). The athlete consumed on average 422 ml of water per hour (range 0 to 1353) and produced 107 ml of urine per hour (range 0 – 650). The athlete lost 0.55 kg of body weight over the duration of the record attempt (72.7 to 72.15 kg).

**Table 1:** Cardiovascular and performance data during the record attempt

**Day**	**Time**	**Distance (km)**	**Speed (km/hr)**	**Heart Rate (bpm)**	**Blood Pressure (mmHg)**
Tuesday	1600–2400	182	22.7	110	124/73
Wednesday	2400–0800	197	24.6	103	146/83
Wednesday	0800–1600	193	24.2	95	134/78
Wednesday	1600–2400	206	25.8	86	135/87
Thursday	2400–0800	193	24.2	76	130/87
Thursday	0800–1444*	155	22.3	76	135/80

	Average	193	24.1	91	134/81
	Total	1126			

*NB the final time period was only 6 hours 44 minutes

**Table 2:** Eight hour (and 24 hour total) energy intake, derived from carbohydrate (CHO), fat and protein, and the calculated energy expenditure during the record attempt

**Day**	**Time**	**CHO (g)**	**Fat (g)**	**Protein (g)**	**Total EI (kcal)**	**Total EE (kcal)**	**EI-EE (kcal)**
Tuesday	1600–2400	383 (87%)	14 (7%)	28 (6%)	1750	4031	-2281
Wednesday	2400–0800	432 (81%)	21 (8%)	59 (11%)	2157	3195	-1038
Wednesday	0800–1600	315 (50%)	114 (39%)	74 (12%)	2590	2749	-159

	**0–24 hour total**	**1130 (73%)** **15.7 g/kg**	**149 (18%)**	**161 (9%)** **2.2 g/kg**	**6497**	**9975**	**-3478**

Wednesday	1600–2400	340 (73%)	38 (17%)	47 (10%)	1892	2040	-148
Thursday	2400–0800	231 (78%)	19 (14%)	21 (7%)	1185	1355	-170
Thursday	0800–1444*	291 (72%)	37 (20%)	31 (8%)	1622	1116	+506

	**24–48 hour total**^#^	**917 (74%)** **12.7 g/kg**	**101 (17%)**	**105 (9%)** **1.5 g/kg**	**5005**	**4722**	**+283**

*NB the final time period was only 6 hours 44 minutes

# extrapolated to 24 hours due to final eight period

**Figure 1:**
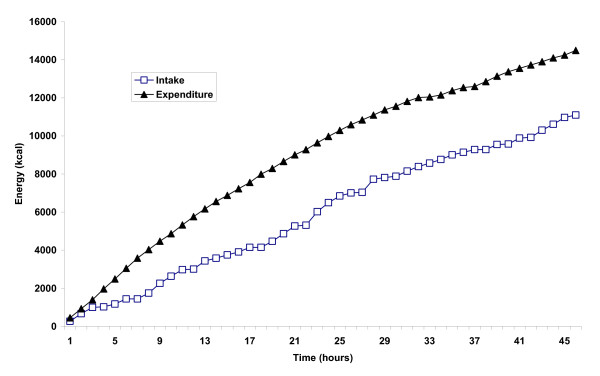
Cumulative energy intake and expenditure across the forty-six hours of continuous cycling.

The cumulative energy intake and expenditure over the entire record attempt are displayed in Figure 1. The total energy intake was 11197 kcal (46.9 MJ) and expenditure 14487 kcal (60.7 MJ), producing an energy deficit over the course of the ride of 3290 kcal (13.8 MJ). The average hourly energy balance was -74 ± 208 kcal (range -543 – 439).

## Discussion

The major finding of this case study was that the athlete was unable to self regulate his energy intake or his exercise intensity to prevent an energy deficit situation.

In comparison to the direct measurement of energy expenditure by calorimetry the individually determined heart rate VO_2 _relationship method, employed in the present study, has been shown to be an acceptable measure of energy expenditure during low intensity exercise [8]. As the energy intake was undertaken by a non-weighed dietary analysis, recorded at the time of consumption, it is possible that the food weights may have been underreported therefore underestimating the total energy intake [9]. Undereating may also result in an energy deficit situation. However the quite remarkable agreement between the estimated energy deficit (3290 kcal) and that calculated from the reduced body mass (3286 kcal)[10] indicates that, on this occasion, the methodologies employed provided an accurate representation of the energy deficit. An alternative explanation is that the methodologies over or underestimated by the same magnitude, which is unlikely given the methodologies for expenditure and intake generally over and underestimate, respectively [11].

The intensity of the ride was 20% higher than required to set the record, therefore unnecessarily increasing the energy expenditure. During the first 24 hour period the athlete's energy expenditure was 9975 kcal (41.8 MJ), less than the 17430 kcal (72.9 MJ) reported during the 2004 XXAlps ultraendurance cycle race [6] or the 23,280 kcal (97.3 MJ) during the 2003 Race Across America (RAAM) [12]. These results, however, were estimated with a Polar S710 heart rate monitor that has been shown to overestimate energy expenditure by 38% compared with the individual relationship between heart rate and oxygen consumption [6]. The energy expenditure during the first 24 hour period compares favourably against values reported during the Tour de France (24.7 – 38.4 MJ) [3,13], cross-country skiing (25.4 – 34.9 MJ) [14], or Artic/Antarctic expeditions (23.6 – 32.4 MJ) [15,16]. The highest recorded daily energy expenditure values were obtained during a trans-Antarctica crossing where for a period of nine days two trekkers averaged 10,564 and 11,634 kcal/d (44.2 and 48.7 MJ/d, respectively) [17], while the theoretical ceiling has been calculated to be 13,675 kcal/d (57.2 MJ/d) [18].

Based on an estimated basal metabolic rate (BMR) for a 70 kg male of 1640 kcal/day [19], the first 24 hour period also represents an expenditure of 6.1 times BMR. Sedentary humans on a daily basis generally expend 1.7 times BMR, while during the 1984 Tour de France cyclists achieved 5.4 times BMR [13]. The highest reported daily expenditure values, 6.7 times BMR, belong to lactating rodents [19]. The results of the current study indicate that the athlete, while exercising at a low intensity (Table 1), did expend an incredible amount of energy over the course of the attempt. The result, however, is inflated in comparison to sustained daily expenditure values [13,19] as the athlete exercised for all but 30 minutes of that 24 hour period.

The athlete averaged a fluid intake of 422 ml/h, nearly identical to the 417 ml/h recorded during a 24 hour road race in Britain [20], but substantially less than the 700 and 1300 ml/h averaged during the RAAM [12] and Tour de France [3], respectively. The fluid intake was also less than the 500–800 ml/h recommended for the cycling leg of Ironman triathlons [21] but considering the extended duration and lower intensity of the exercise the self-regulated fluid intake appeared to be appropriate to maintain euhdryation.

The total volume of fluid consumed, plus the water contained within food, was 13 litres greater than the volume of urine produced. This positive balance did not result in an increase in unexplained body mass as the intensity of exercise maintained has been shown to produce sweat at a rate of 233 ml/hour [22], and insensible water loss and faeces, while not measured, could have accounted for the unexplained 2.4 litres. Ultraendurance exercise has also been shown to increase total body water [23] and it is possible that the loss of water due to sweat may be less than estimated, as the rate of sweat production would have fluctuated with the changing climatic conditions particularly overnight.

The minimal sweat rate maintained throughout the attempt would also have enabled the athlete to reabsorb, within the sweat duct, the majority of the sodium secreted by the gland. In more humid and hot conditions, and in unacclimatised individuals, the increased risk of exercise associated hyponatremia [24] resulting from minimal sodium ingestion or excessive water intake, can result in complications that are potentially life-threatening [25]. The American College of Sports Medicine therefore recommends sodium supplementation at a rate of 0.5–0.7 g/L (8.6–12 mmol/L) in their position stand on fluid replacement for events lasting more than one hour [26]. In longer ultraendurance events the risk of hyponatremia is substantially increased and supplementation rates of up to 1.15 g/L (50 mmol/L) have been proposed for events lasting 24 hours [27,28]. In the current case study the athletes sodium intake of 0.008 g/L (0.03 mmol/L) was significantly below the recommended levels and, regardless of his calculated sweat rate resulting in minimal sodium loss, may have resulted in the athlete suffering from hyponatremia as evidenced by the athlete's inability to stand, dizziness, and confused state upon retiring from the attempt.

The current recommendations for CHO supplementation during ultraendurance exercise range from 30 – 90 g/h [21,27,29]. The modality, running [29] compared with cycling [21], and the definition of ultraendurance, hours [21,29] compared with days [27], could explain the substantial range prescribed. During the current study the athlete averaged only 42 g/h and consumed greater than the minimum recommended CHO dose only 52% of the time. CHO is essential in prolonged low intensity exercise in order to provide essential intermediaries (ie oxaloacetate and pyruvate) for fat oxidation. The levels of CHO intake, in the current study, may have resulted in depletion of endogenous stores and subsequent hypoglycaemia. Sufficient CHO intake is necessary to maintain exercise for prolonged periods without the onset of fatigue [30].

Despite being provided with appropriate guidelines, the athlete did not pre-plan or prepare his energy intake for the record attempt. He supplemented his intake of sports drinks, protein bars, and energy gels (Endura™) with impulse selections from nearby commercial food outlets. These impulse selections included fried rice, McDonalds™, and bacon and eggs. In any ultraendurance event, requiring the athlete to eat while competing, it would be strongly recommended that the athlete commence the event with all meals and fluids planned and prepared according to nutritional recommendations, likes and dislikes, and ability to store items to be consumed. Alternatives also need to be prepared for when food and flavour fatigue occurs, as is often the case during ultraendurance events (unpublished – personal observation). Poor planning and preparation, as well as other common factors such as gastrointestinal discomfort [1] and loss of appetite [4], may be reasons for the athlete failing to match his energy requirements.

It is unknown from the current data exactly why the athlete was unable to complete the record attempt. An energy deficit situation, and therefore loss of body mass, is common in ultraendurance events [6,16,20,27,31] and therefore cannot directly account for the failed attempt. The insufficient CHO intake may have contributed, as it has previously been correlated with poor performance [31] and associated with the onset of fatigue [30] in ultraendurance triathlon. Aside from the insufficient CHO intake, the lack of sleep and a compromised immune system due to an undisclosed viral complaint, could have potentially contributed to the failed attempt. It should be noted however that the athlete had had previous experience at ultraendurance races that involved sleep deprivation and his preparation for this event was similar.

## Conclusion

The initial eight hour period particularly highlights the inadequacies of self-regulation with a lower than required total energy, and particularly CHO, intake, and an elevated energy expenditure. Overcoming such a large initial energy deficit, while the athlete is still competing, is difficult due to the limited digestive capacity of the gastrointestinal tract [3]. The inability for the athlete to balance their own energy requirements reflects the importance of the role(s) played by the sports dietitian and exercise physiologist in planning and monitoring the energy intake and expenditure, respectively, for ultraendurance performance.

## Authors' contributions

IBS conducted the laboratory testing, physiological data analysis and drafted the manuscript.

KLS conducted the nutritional data analysis and contributed to the manuscript.

All authors read and approved the final manuscript.
